# Stapled diverticulectomy for solitary caecal diverticulitis

**DOI:** 10.1308/003588412X13373405387131

**Published:** 2012-05

**Authors:** RU Uwechue, ER Richards, M Kurer

**Affiliations:** Scarborough and North East Yorkshire Healthcare NHS Trust,UK

**Keywords:** Caecal diverticulitis, Laparoscopic, Diverticulectomy

## Abstract

Caecal diverticulitis is an uncommon phenomenon in western countries. The clinical diagnosis is often difficult as it mimics other acute abdominal conditions like appendicitis, colitis or neoplasia. Diagnosis is often made at operation. Operative strategy has been controversial and there is no broad consensus emerging. We report the case of a 71-year-old woman, known to have chronic obstructive pulmonary disease, who presented acutely with right iliac fossa pain. A clinical diagnosis of appendicitis was made. At laparoscopy, a solitary, inflamed, gangrenous caecal diverticulum was found. A laparoscopic stapled diverticulectomy was performed. The patient made a steady post-operative recovery. Histology confirmed diverticulitis. We conclude that stapled diverticulectomy for solitary caecal diverticulitis is a safe and effective surgical strategy when confronted with this scenario.

Caecal diverticulitis was first described by Potier in 1912. It is a well recognised pathological entity yet it is still uncommon in Western society. It has a higher prevalence in Asian populations.^1^

Presentation is commonly with a history of right-sided abdominal pain and a clinical examination indistinguishable from appendicitis. Caecal diverticulitis is frequently diagnosed at laparoscopy or laparotomy and various treatment options have been described in the literature.

## Case history

A 71-year-old woman was admitted to our hospital complaining of several days of acute onset abdominal pain. Her pain started as a dull central ache that had migrated to the right lower quadrant. She was known to have chronic obstructive pulmonary disease (COPD) and her exercise tolerance was significantly restricted. Examination revealed tenderness and guarding in the right iliac fossa, over McBurney’s point. Her inflammatory markers were raised, her total white cell count was 11.8 × 10^9^/l (normal range: 4–11 × 10^9^/l) and her C-reactive protein was 116mg/l (normal range: <6mg/l). A clinical diagnosis of appendicitis was made and she was taken to the operating theatre for a diagnostic laparoscopy.

**Figure 1:**
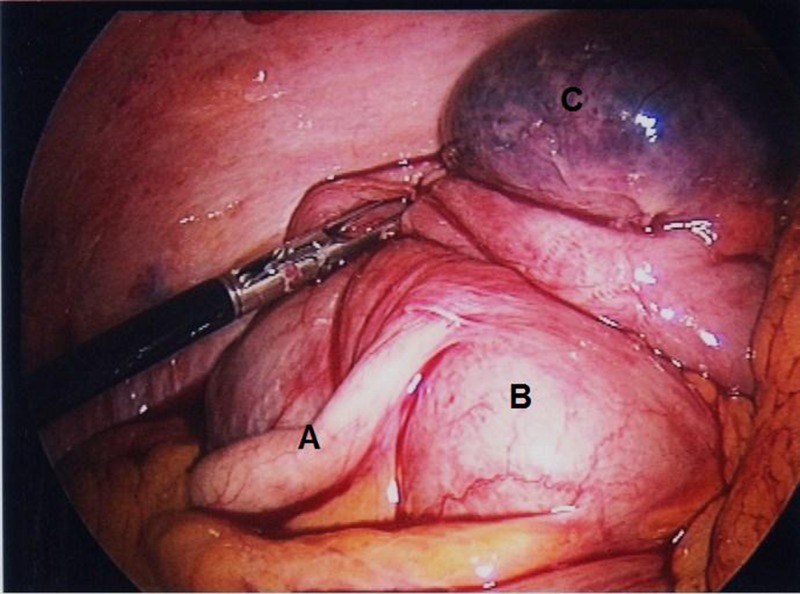
Solitary caecal diverticulitis with normal appendix

**Figure 2:**
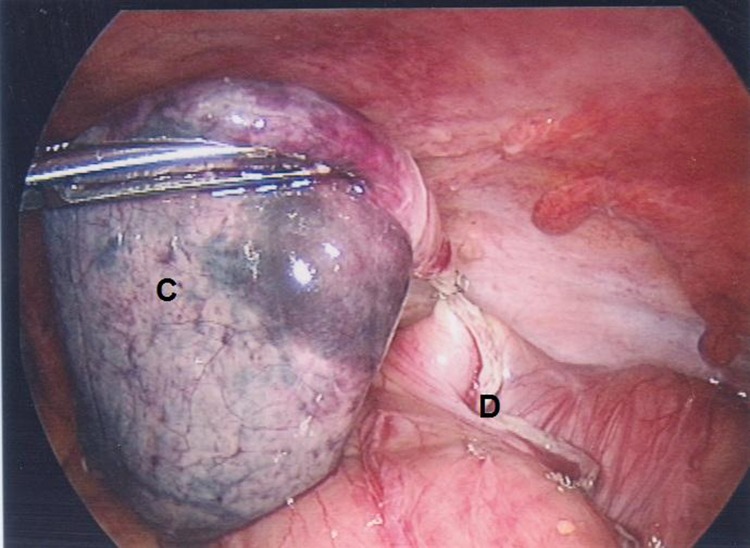
Diverticulum partially excised

**Figure 3:**
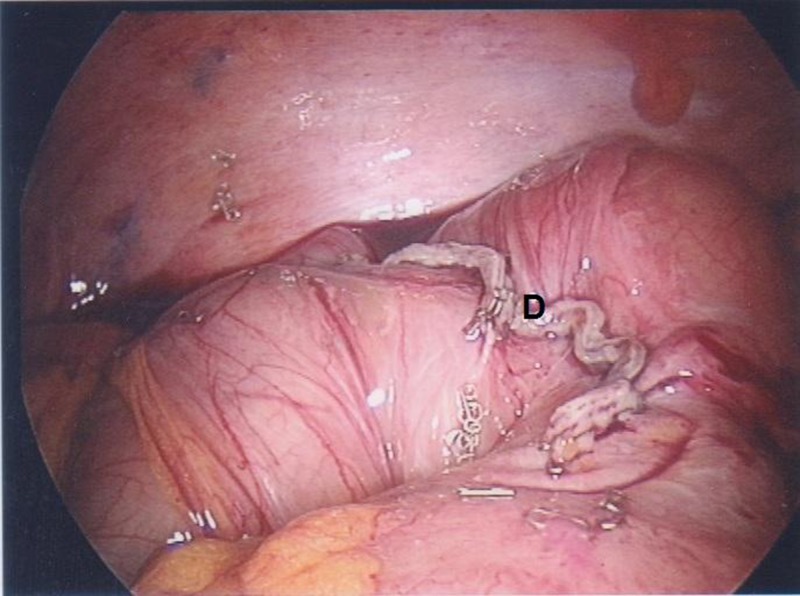
Staple line following resection

A 10mm umbilical balloon port was inserted by open cut-down technique. A left iliac fossa 12mm port and a suprapubic 5mm port were inserted under vision. The appendix was normal and an inflamed and gangrenous solitary diverticulum was noted on the antimesenteric surface of the caecum (Fig 1). In view of her co-morbidities, the patient was not felt to be fit for open surgery or an extensive resection. A stapled diverticulectomy was therefore performed using an Endo GIA™ (Covidien, Dublin, Ireland) stapling device (Figs 2 and 3).

Histology confirmed a 3.5cm × 3.0cm × 1.5cm diverticulum with transmural suppurative inflammation and loss of the mucosal lining. The patient made a slow recovery from surgery. She developed a post-operative chest infection that was treated successfully. She was discharged a week after surgery. At the follow-up appointment two months later, she had made a full recovery. She had developed a port site hernia at the left iliac fossa port but was asymptomatic from this.

## Discussion

The diagnosis of caecal diverticulitis is difficult to establish accurately pre-operatively. The clinical presentation often mimics that of acute appendicitis as seen in this case. Consequently, the diagnosis is frequently made at the time of surgery for presumed appendicitis.^1^

Treatment options for caecal diverticulitis vary widely depending on the presentation and local expertise. There is no consensus among surgeons as to the best option. For uncomplicated caecal diverticulitis diagnosed pre-operatively, a conservative approach can be taken with bowel rest and antibiotics.^2^ However, others advocate aggressive surgical resection in caecal diverticulitis as less than 40% of patients are successfully managed conservatively without recurrent symptoms.^3^

The surgical approach to resection of a solitary caecal diverticulum varies from a simple diverticulectomy to a right hemicolectomy. These can be performed open or laparoscopically. The first report of a laparoscopic diverticulectomy was in 1994.^4^ Since then, there have been several other reports showing that laparoscopic resection is feasible in experienced hands.^1^

Our case posed several challenges. First, the patient had COPD, a significant medical co-morbidity. This increased her risk of peri-operative and post-operative complications. It was therefore felt that laparoscopic resection was the most appropriate option in order to reduce these risks as it involved smaller incisions and facilitated faster recovery. Indeed, the patient did suffer from a post-operative chest infection, which was treated successfully.

Second, the diagnosis was made intra-operatively and a decision as to how to treat this needed to be made intra-operatively. Resection was performed as the diverticulum was gangrenous and perforation was thought to be imminent, the risk of recurrence if left unresected is known to be significant^3^ and the surgeon was happy to proceed based on his experience.^5^ Furthermore, the diagnosis of caecal cancer must always be entertained in all acute abdomens with intra-operative caecal pathology. If suspected, an oncologically sound right colectomy is the treatment of choice. In reference to our case and as is clear from the pictures, we felt cancer was unlikely. This was because we encountered a discrete, well defined, outpouching area of the caecal wall (the solitary caecal diverticulum), which looked gangrenous, and felt rather soft and as though it were about to perforate. We were confident that no characteristics of malignancy were present.

Finally, the patient developed a port site hernia at the 12mm left iliac fossa incision. We feel that this is most likely a result of the patient’s chronic cough that she experiences as part of her COPD. This highlights the importance of careful consideration of port placement and closure technique, taking into account the general condition of the patient. This port site was not sutured at the sheath. In hindsight, it may have been prudent to have closed the sheath.

## Conclusions

A laparoscopic stapled diverticulectomy for solitary caecal diverticulitis for a safe and effective therapeutic option, especially in patients in whom more radical surgery would pose a high risk. We believe this is the first reported case in the UK.
